# Small average differences in attenuation corrected images between men and women in myocardial perfusion scintigraphy: a novel normal stress database

**DOI:** 10.1186/1471-2342-11-18

**Published:** 2011-10-03

**Authors:** Elin Trägårdh, Karl Sjöstrand, David Jakobsson, Lars Edenbrandt

**Affiliations:** 1 Clinical Physiology and Nuclear Medicine Unit, Skåne University Hospital, Lund University, Malmö, Sweden; 2 Informatics and Mathematical Modeling, Technical University of Denmark, Copenhagen, Denmark

## Abstract

**Background:**

The American Society of Nuclear Cardiology and the Society of Nuclear Medicine state that incorporation of attenuation-corrected (AC) images in myocardial perfusion scintigraphy (MPS) will improve image quality, interpretive certainty, and diagnostic accuracy. However, commonly used software packages for MPS usually include normal stress databases for non-attenuation corrected (NC) images but not for attenuation-corrected (AC) images. The aim of the study was to develop and compare different normal stress databases for MPS in relation to NC vs. AC images, male vs. female gender, and presence vs. absence of obesity. The principal hypothesis was that differences in mean count values between men and women would be smaller with AC than NC images, thereby allowing for construction and use of gender-independent AC stress database.

**Methods:**

Normal stress perfusion databases were developed with data from 126 male and 205 female patients with normal MPS. The following comparisons were performed for all patients and separately for normal weight vs. obese patients: men vs. women for AC; men vs. women for NC; AC vs. NC for men; and AC vs. NC for women.

**Results:**

When comparing AC for men vs. women, only minor differences in mean count values were observed, and there were no differences for normal weight vs. obese patients. For all other analyses major differences were found, particularly for the inferior wall.

**Conclusions:**

The results support the hypothesis that it is possible to use not only gender independent but also weight independent AC stress databases.

## Background

Stress myocardial perfusion scintigraphy (MPS) is widely regarded as a clinically useful noninvasive imaging modality for diagnosing patients with suspected coronary artery disease [1-3]. However, Compton scatter and depth-dependent reduction of spatial resolution degrade MPS image quality and decrease test accuracy. In addition, localized soft-tissue attenuation by the breasts, lateral chest wall, and abdomen may create artefacts that mimic true perfusion abnormalities and decrease test specificity [4,5].

Several studies have reported an increase in the diagnostic accuracy (through higher specificity) for the detection of coronary artery disease when MPS is attenuation corrected (AC) [6-12]. The American Society of Nuclear Cardiology and the Society of Nuclear Medicine conclude in their joint position statement from 2004 [6] that incorporation of AC in addition to ECG gating with MPS images will improve image quality, interpretive certainty, and diagnostic accuracy. These combined results are anticipated to have a substantial impact on improving the effectiveness of care and lowering health care costs. However, commonly used software packages for MPS usually only include a normal stress database for non-attenuation corrected (NC) images.

The three most commonly used software packages for viewing and quantifying MPS are Emory Cardiac Toolbox (Emory University), 4D-MSPECT (Invia Medical Imaging Solutions) and Quantitative Perfusion SPECT (Cedars Sinai). The Emory Cardiac Toolbox uses 3 populations for generating a normal database [13]: (1) a normal group of 30 patients with a low likelihood of coronary artery disease; (2) a criteria group of 60 patients with different perfusion abnormalities; and (3) a prospective validation group of 60 patients. This is done for both male and female patients. A second step is used to calibrate regionally how many standard deviations below the mean best separate normal from hypoperfused segments. The 4D-MSPECT uses a normal database comprising patients with a low pretest likelihood of coronary artery disease [14]. The database has more than 40 patient studies. The Quantitative Perfusion SPECT software also creates normal limits by use of low likelihood patients [15].

The aim of the present study was to develop and compare different normal stress databases for MPS with respect to both NC and AC images and with respect to both gender and the presence vs. absence of obesity. The principal hypothesis was that AC images show smaller differences between men and women compared with NC images, thus allowing for a gender independent AC normal stress database. Previous studies with the same aim [16-18] have all used a different technique than the one described herein for selecting the patients for the normal stress database. Our approach is most applicable to hospitals desiring to create normal stress databases for MPS that are optimized to their own patient populations.

## Methods

### Study population

Patients who underwent 99mTc MPS at Skåne University Hospital in Malmö, Sweden, in 2008, for suspected coronary artery disease or for the management of known coronary artery disease, were considered for inclusion. The study was approved (# 647/2008) by the ethics committee on human research at Lund University, Sweden, and complies with the Declaration of Helsinki. The normal population was found using the following steps:

1) Patients were included if they had a normal test result, meaning neither fixed nor reversible perfusion defects, normal ejection fraction (≥ 60% for women, ≥ 55% for men) and normal end diastolic volume (≤ 132 ml for women, ≤ 181 ml for men) [19] based on the final report according to clinical routine. Both NC and AC images were used for interpretation. The physicians who interpreted the images had access to all patient history and exercise protocol, but were not aware that the patients might be included in a study. The EXINI heart™ (EXINI Diagnostics AB, Lund, Sweden) software package was used for interpretation [20]. This software displays MPS images, quantifies perfusion defects and cardiac function, and gives diagnostic advice based on artificial neural networks.

2) From the group with normal findings in step 1, patients with documented diabetes, coronary artery disease, previous myocardial infarction, previous revascularization, electrocardiographic signs of myocardial infarction (based on computer interpretation), presence of pre-excitation, paced rhythms and left bundle branch block were excluded. After this, 131 men and 213 women were included.

3) Bull's eye plots for these patients were created, and obvious 'non-normal' patients were excluded (5 men and 8 women). Finally, 126 men and 205 women were included in the study group. These patients were further divided into one group of normal weight and one group of obese (body mass index (BMI) > 30 kg/m^2^) patients.

### MPS

The MPS studies were performed using a 2-day gated stress/non-gated rest Tc-99m-tetrofosmin protocol, starting with injection of 600 MBq Tc-99m-tetrofosmin at stress. Patients were stressed using either maximal exercise on an ergometer or pharmacological test with adenosine. The exercise was continued for at least 1 min after the injection of the tracer and the adenosine infusion at least 2 min after the injection of the tracer. Normal findings at stress were not followed by a rest study. Not definitely normal stress studies were followed by a rest study with injection of 600 MBq Tc-99m-tetrofosmin.

Stress and rest acquisition began about 60 min after the end of the injection of Tc-99m-tetrofosmin. Images were obtained according to established clinical protocols, using SPECT over 180° elliptical, autocontour rotations from the 45° right anterior oblique position, with a dual-head gamma camera, e.cam (Siemens AG Medical Solutions, Erlangen, Germany). Patients were imaged in the supine position. Low energy high-resolution collimator and a zoom factor of 1.0 were used with 64 (32 views per camera) projections in a 128 × 128 matrix and an acquisition time of 25 s per projection. Stress images were gated to the electrocardiogram using 8 frames per cardiac cycle. No automatic motion-correction program was applied; instead the acquisition was repeated if motion was detected. This was decided by the physician in charge of the study, based on qualitative criteria. Tomographic reconstruction and calculation of short and long axis slice images were performed using e.soft (Siemens AG Medical Solutions, Erlangen, Germany). NC images were reconstructed with filtered back-projection. A 2D Butterworth pre-reconstruction filter was used with clinical frequency of 0.45, order 5. AC images were reconstructed with an iterative algorithm, 6 iterations [21] where a ramp filter was applied on the error projection prior to backprojection. A Butterworth filter with a clinical frequency of 0.40, order 5, was applied for regularization. Attenuation maps were generated from simultaneous transmission measurement using a Gd-153 multiple-line source (Siemens AG Medical Solutions, Erlangen, Germany) [22]. The cut-off frequencies of the filters were selected so that the noise level in the AC images was similar to that in the NC images.

### Image processing

Normal stress perfusion databases were developed as follows: Each normal stress map was scaled such that its median value, in the region or regions including the 10% pixels with highest count values was made equal. This normalization makes the stress data equal in a region that is likely to show the most normal perfusion.

### Statistical analysis

The comparison method consisted both of a pixel-by-pixel analysis and a segmental analysis. The following tests were carried out for all patients, for patients with normal weight, and for obese patients: NC men vs. NC women; AC men vs. AC women; NC men vs. AC men; and NC women vs. AC women. For all patients, also NC normal weight vs. NC obese; and AC normal weight vs. AC obese were carried out. For all women and all men, the following tests were carried out: NC normal weight vs. NC obsese; and AC normal weight vs. AC obese. Groups where compared using two-sample t-tests for men vs. women comparisons, and paired t-tests for AC vs. NC comparisons. The level of statistical significance was set at α = 0.001. The database images are of size 65-by-65 pixels yielding a total of 4225 tests for each comparison. Due to random components in the images such as noise, many of these tests may be rejected also when the null hypothesis (no difference) is true everywhere. To adjust for this effect a non-parametric permutation scheme outlined in [23] was employed. The method proceeds by computing pixel-wise differences between groups when the group assignment for the images is perturbed. For such datasets, one does not expect significant differences, and we record the maximal absolute t-value as a measure of the most extreme outcome given no differences. This procedure is repeated many times (here, 500), yielding an empirical distribution of maximal t-values. The original (unperturbed) t-value image is then thresholded at the t-value corresponding to the 100*(1 - α)th percentile of the maximal t-value distribution. This procedure provably maintains strong control over type-I errors, meaning that the probability of declaring a difference at any pixel when in fact there is none is at most α. Furthermore, the probability of falsely declaring a difference at a specific pixel, regardless of any true difference elsewhere, is at most α.

The area for the different segments was created in the following way: The apical segment was delineated from apex to 30% of the distance to the base. The rest of the area was divided into four equally sized areas: lateral, inferior, septal and anterior, without overlap. The most basal 10% were not included into the segmental analysis in order to avoid differences in perfusion pattern due to differences in the basal delineation of the left ventricle.

## Results

### Description of patients

For the 205 women, mean age was 59 years (ranging from 29 to 87 years). For the 126 men, mean age was 56 years (ranging from 27 to 86 years). Forty (40) women were regarded as obese (mean BMI 34.0 kg/m^2^) and 165 women had normal body weight (mean BMI 24.6 kg/m^2^). For men, 24 were obese (mean BMI 33.1 kg/m^2^) and 102 had normal weight (mean BMI 25.5 kg/m^2^).

### All patients

**Figure 1:**
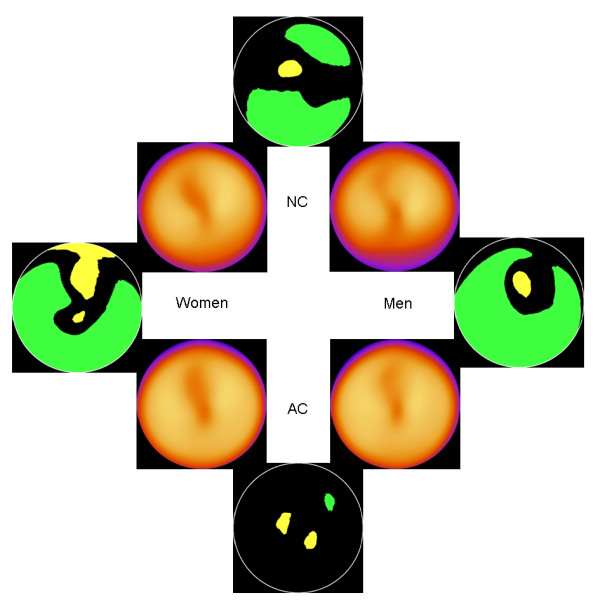
**All patients**. The 4 inner images show the normal stress databases for NC men, NC women, AC men and AC women. The 4 outer images show areas with statistically significant differences. Green colour means significantly lower counts for men or for NC images, depending on comparison, and yellow colour means significantly higher counts.

Figure 1 shows the normal stress data base for AC men, AC women, NC men and NC women, respectively. Included also are the areas with a significant difference (green or yellow) in mean counts. In the AC databases, when comparing men and women, only very small areas of the bull's eye plot show a statistically significant difference, whereas there were large areas of significant differences within the other groups tested. When comparing women within the NC and AC databases, the largest difference in mean counts was found in the inferior wall (lower in the NC group). The same result was found in men. For men and women in the NC group, men showed lower mean counts in the inferior wall. When comparing men and women in the AC group, the largest differences were found in small areas in the apex and lateral wall. Table 1 shows differences in mean count values (± 2 standard deviations), from a scale ranging from -100 to 100 (0 meaning no difference), in different groups for different segments.

**Table 1:** Differences in mean count values (± 2 standard deviations), from a scale ranging from -100 to 100 (0 meaning no difference), in different groups for different segments

	Women (NC/AC)	Men (NC/AC)	NC (Women/Men)	AC (Women/Men)
**Apical**	1.5 (± 3.9)	1.0 (± 6.2)	2.1 (± 6.6)	2.1 (± 2.4)
**Lateral**	-3.9 (± 8.5)	-4.1 (± 9.6)	-1.6 (± 4.0)	-2.1 (± 3.3)
**Inferior**	-7.4 (± 8.4)	-14.0 (± 7.5)	-6.6 (± 4.4)	-0.6 (± 3.5)
**Septal**	-5.9 (± 5.0)	-7.6 (± 7.5)	-0.7 (± 6.4)	0.5 (± 3.2)
**Anterior**	2.4 (± 6.0)	0.4 (± 7.1)	-1.6 (± 6.3)	-0.2 (± 4.0)

### Patients divided into groups based on BMI

**Figure 2:**
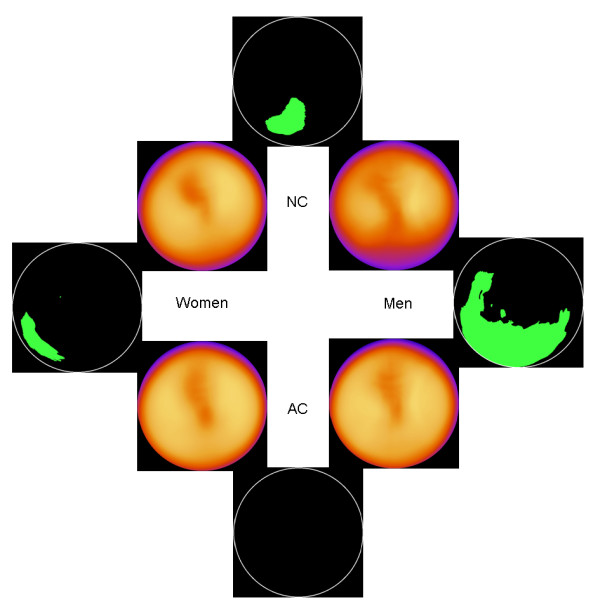
**Patients with normal weight**. The 4 inner images show the normal stress databases for NC men, NC women, AC men and AC women. The 4 outer images show areas with statistically significant differences. Green colour means significantly lower counts for men or for NC images, depending on comparison, and yellow colour means significantly higher counts. There are no significant differences between AC men and AC women (bottom black circle).

Figure 2 shows the normal stress databases and the statistically significant differences between them, when only including patients with BMI < 30 kg/m^2^. In the AC database, when comparing men and women, no statistically significant differences were found. When comparing NC women with AC women, mean counts were lower in the NC women in the basal inferolateral wall. For the other groups (NC women vs. NC men and NC men vs. AC men) differences were found in the inferior wall.

**Figure 3:**
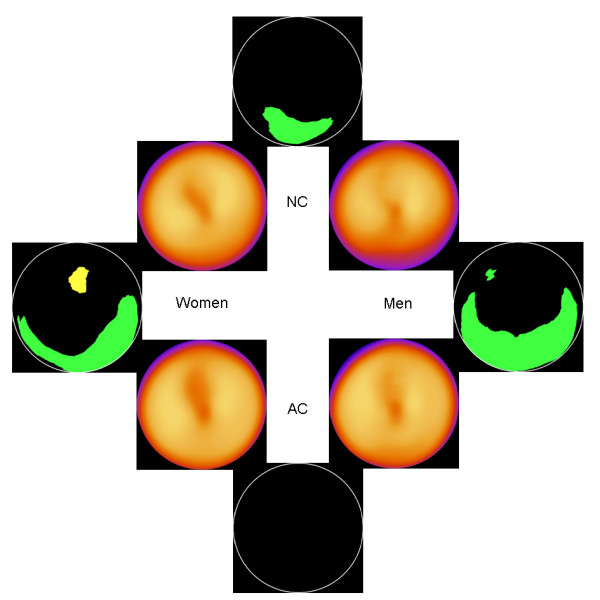
**Obese patients**. The 4 inner images show the normal stress databases for NC men, NC women, AC men and AC women. The 4 outer images show areas with statistically significant differences. Green colour means significantly lower counts for men or for NC images, depending on comparison, and yellow colour means significantly higher counts. There are no significant differences between AC men and AC women (bottom black circle).

Figure 3 shows the normal stress databases and the significant differences between them, for patients with BMI > 30 kg/m^2^. Also for these patients, no statistically significant differences were found when comparing mean counts between AC women and AC men. For all other analyses (NC women vs. AC women, NC men vs. AC women, NC women vs. NC men), there were significant differences in the inferior wall.

**Figure 4:**
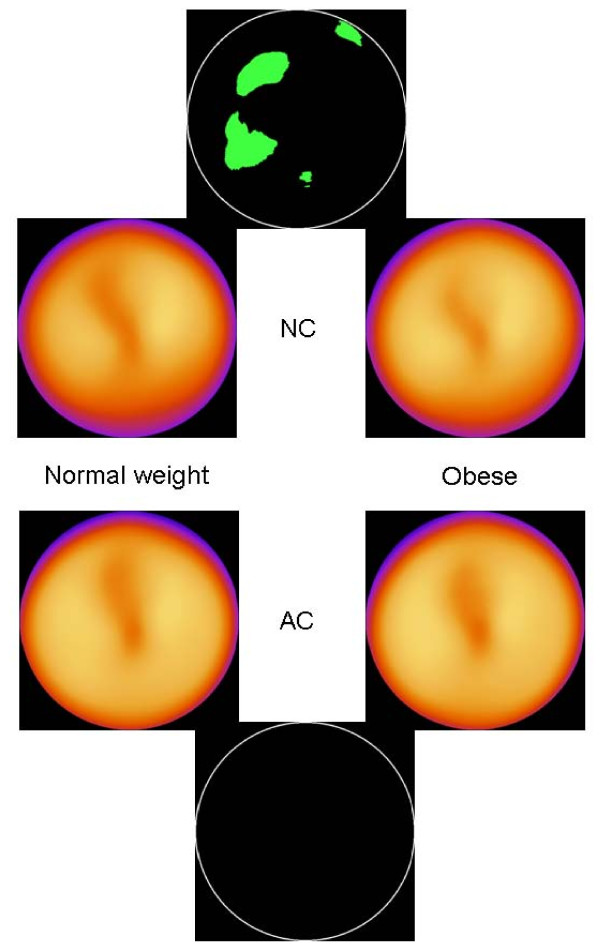
**All patients**. The 4 inner images show the normal stress databases for NC normal weight individuals, NC overweight individuals, AC normal weight individuals and AC overweight individuals. The 2 outer images show areas with statistically significant differences. There are no significant differences between AC normal weight and AC overweight individuals (bottom black circle).

When comparing NC normal weight men with NC obese men, no statistically significant differences was found. This was also true when comparing AC normal weight men with AC obese men, and AC normal weight women with AC obese women. When comparing NC normal weight women with NC obese women, 0.1% of the total area (inferoseptal, midventricular wall) had statistically different mean count values between the groups (data not shown). When instead dividing all patients into groups of NC normal weight, NC obese, AC normal weight and AC obese (no gender separation) we found significant differences between NC normal weight and NC obese individuals, but no differences between AC normal weight and AC obese individuals (Figure 4).

## Discussion

We compared NC and AC normal stress databases for men and women and demonstrated that there were significant differences in mean counts in large areas of the left ventricle when comparing NC men and NC women, as well as when comparing NC men and AC men, and NC women and AC women. The differences were much smaller when comparing AC men and AC women. No significant differences were found when comparing AC normal weight and AC obese individuals, whereas there were significant differences when comparing NC normal weight and NC obese individuals. Thus, we have demonstrated that AC eliminates the differences between gender and body weight, which supports the hypothesis that it is possible to use a gender independent and body weight independent AC stress database. Use of such a database would be favourable because only one stress database therefore becomes necessary and nuclear cardiologists need not learn to distinguish between different possible attenuation artefacts.

The reason for the small areas of statistical difference between AC men and AC women (all patients), compared to no statistical difference when comparing AC men and AC women (normal weight patients and obese patients) was probably due to the first comparison being less susceptible to type-II statistical error given the larger number of individuals involved.

A previous study by Grossman et al [16] investigated the accuracy of an AC database quantification program for the detection and localization of coronary artery disease and evaluated its application to a heavy patient population as compared with standard uncorrected methods. They found no statistically significant differences when comparing AC perfusion distributions of normal men and women, whereas significant differences were found in the same NC studies. They also found that AC improved quantitative analysis, yielding a significantly higher normalcy rate and specificity without a significant loss in sensitivity, even in an obese patient population. In their study, only 26 men and 22 women were included in the normal group (compared to 126 men and 205 women in our study). They also did not perform any studies on the differences between overweight patients and patients with normal weight for the normal stress database. Previous studies by Ficaro et al [17] and Slomka et al [18] also included a lower number of patients in the normal stress database (20 men and 20 women; 50 men and 50 women, respectively). Ficaro et al [17] found no significant differences between the male and female AC distributions, whereas Slomka et al [18] noted some gender differences for the AC normal database. Slomka et al [18] also investigated body mass index-specific normal limits, and found no significant differences between normal studies of low-BMI and high-BMI patients for either the AC or NC data set. None of these previous studies have used a pixel-by-pixel analysis, but instead only examined segmental differences in the 17-segment model.

The pre-installed databases of normal values in commercial software programs are generally based on the results obtained in a U.S. population, and may not be optimal for assessing a population form another country. It has previously been shown that there are major differences between US and Japanese normal databases in nuclear cardiology [24], particularly in the apex and in the anterior wall in females and in the inferior wall in males. Similar results have been found in a Chinese [25] as well as a Spanish population [26]. We believe that in an ideal situation, every clinic should create its own normal stress database, instead of using the ones offered by the company providing the imaging evaluation software. Different cameras with different techniques used (for example filtered backward projection or iterative reconstruction) produce images that may not be directly comparable to the normal stress database included in the software nor to the distribution of body habitus in the population. Creating normal databases, however, consumes time and resources. Properly implemented AC methods should remove differences in the normal perfusion distribution that are due to variations in body habitus including gender, as shown in the present paper. For clinics using AC methods, a gender independent and body-weight independent stress database is easier to create than to produce separate databases for male and female subjects with different weights.

In this study a Gd-153 transmission source was used for attenuation correction. In the computed tomography - single photon emission computed tomography hybrid devices, computed tomography-based correction may perform differently than a radionuclide-source correction. We believe that methods for attenuation correction other than Gd-153 also will also reduce the influence on gender and body habitus on MPS images, as indicated by other studies [13-15], but when a different technique is used, it is probably necessary to create a new normal stress database.

Further studies are needed to evaluate the accuracy of the image interpretation when using a gender- and body weight independent normal stress database such as the one described herein.

### Study limitations

One study limitation is that the included individuals were not a random sample from the healthy population, but instead "healthy patients". Another limitation is that the number of obese patients was low compared to patients with normal weight. For the selection of the normal population, only MPS was used and not any other imaging modality. If coronary angiography was used to select normal patients, patients with a "non-normal" MPS, but a normal coronary angiography, could have been included in the normal database. It might be that "pseudo-pathological" MPS are excluded from our study, which ought to be included. However, if we had required a normal coronary angiogram for inclusion, patients with microvascular disease might have been included in the normal MPS stress database, which would have been equally problematic.

## Conclusion

In conclusion, differences in mean counts when comparing men and women in the AC group were much smaller than when comparing the other groups; no differences were found in this group when dividing patients according to body weight. The results support the hypothesis that it is possible to use gender independent and body weight independent AC stress databases. Use of such databases would be favourable because only one database therefore becomes necessary and nuclear cardiologists need not learn to distinguish between different possible attenuation artefacts.

## List of Abbreviations Used

AC: attenuation-corrected; BMI: body mass index; MPS: myocardial perfusion scintigraphy; NC: non-attenuation-corrected

## Declaration of Competing interests

LE, KS and DJ are employed by and stockholders of EXINI Diagnostics, Lund, Sweden.

## Authors' contributions

ET participated in the design of the study, the selection of patients and drafted the manuscript. KS and DJ performed the statistical analyses, image processing and helped in drafting the manuscript. LE participated in the design of the study and helped in drafting the manuscript. All authors read and approved the final manuscript.

## Pre-publication history

The pre-publication history for this paper can be accessed here:

http://www.biomedcentral.com/1471-2342/11/18/prepub
